# Trained Army Nurses in Colonial India: Early Experiences and Challenges

**DOI:** 10.1017/mdh.2023.31

**Published:** 2023-10

**Authors:** Preethi Mariam George, John Bosco Lourdusamy

**Affiliations:** Department of Humanities and Social Sciences, Indian Institute of Technology Madras, Chennai-600036, Tamil Nadu, India

**Keywords:** Military Nursing, Female Agency, Nursing Sisters, Colonial India, Indian Nursing Service, Catharine Grace Loch

## Abstract

The paper examines the introduction of trained female nurses for the British army men in colonial India between 1888 and 1920. It discusses the genesis of the Indian Nursing Service (INS), including the background and negotiations leading up to its formation, terms of employment, duties and working conditions of the nursing sisters. The memoir of Catharine Grace Loch, who served as the first Chief Lady Superintendent of the service is used extensively to trace the early experiences and challenges of the nursing sisters. The paper primarily argues that the INS being a new service, the colonial government maintained tight control over its functioning, and extreme conservatism in spending, thus retarding the growth of professional army nursing in India. Secondly, in examining the relations between the sisters and the (male) nursing orderlies, sub-medical and medical officers, the paper argues that the inadequate delineation of the nursing sisters’ position in the military medical hierarchy was an important reason for the undermining of their expertise and status. Thirdly, the paper contends that as an all-women service, nursing constituted an important avenue of female agency within the patriarchal colonial establishment, which subjected the sisters to scrutiny both professionally and socially. The paper analyses the resultant conditions and regulations imposed on the sisters – most of them determined by gender and class notions. Finally, the paper discusses the gradual establishment and recognition of the service as an important cornerstone for the health of the army, while highlighting the shortcomings that yet persisted up until 1920.

## Introduction

The advent of western medicine in India was inextricably linked to the colonial requirement to cater to the health of the British population, especially the army, which was adversely affected by a variety of diseases encountered in India. There is a rich and diverse scholarly engagement with several dimensions of medicine and health in the Indian colonial context.^1^ But within this, the question of nursing, an important element of western medical practice, has not received commensurate scholarly attention except for a very few works.^2^ This paper aims to analyse some aspects of this much neglected domain by focusing on the early years of the introduction of trained nurses for the British army in India.

In Victorian England, the care of the sick was conventionally regarded as one among the feminine responsibilities.^3^ Women who undertook nursing, both inside and outside the domestic sphere, were untrained. In the nineteenth century, reforms in the hospital and ward system, along with the development of specialised treatment methods created a need for trained nurses.^4^ Nursing reforms gained traction by the second half of the nineteenth century, under the leadership of Florence Nightingale and others. Nightingale insisted on a systematic course for training women as skilled nurses, while maintaining that only women had the aptitude and competence for care that required gentle handling and housekeeping skills.^5^ The reforms gradually resulted in the professionalisation and feminisation of nursing.

With regard to the army, the nursing functions were predominantly performed by male orderlies until the mid-nineteenth century. During the Crimean War that began in 1853, the British army lost an alarming number of its soldiers due to insanitary conditions and diseases. Under such circumstances, Nightingale, in 1854, pioneered the work of providing organised female nursing to the British army at Scutari during the course of the war.^6^ She went on to become an ardent advocate of the employment of trained female nurses, especially as a major step towards improving the health of the army. Nightingale envisioned female nurses in military hospitals to have the power to give orders to the male ward orderlies, and ensure the obedience and discipline of the male soldiers admitted to the wards for treatment.^7^ The idea of female nurses was initially resisted based on the assumption that female presence would ‘excite the passions’ of men. In partial concession to this concern, Nightingale recommended that the nurses attend only to the severely ill or injured.^8^ They were not to be employed in wards of patients with venereal diseases or for patients who were just convalescing. Based on these suggestions and recommendations, female nurses came to be employed in the military hospitals in Britain.

In colonial India, nursing was not undertaken by professionally qualified and certified nurses, in either civil or army settings, until the second half of the nineteenth century. Nuns and missionaries offered nursing care to the ailing natives as part of Christian medical mission work. Following nursing reforms in England, there were sporadic attempts to provide trained nurses for government civil hospitals. In this process the government relied heavily on philanthropic efforts.^9^ In 1865, Nightingale suggested that a few matrons and head nurses should be sent from England to start training of nurses in civil hospitals. But the colonial government rejected this modest plan on the ground that it was expensive. However, Lord Napier, the Governor of Madras Presidency, who was well acquainted with Florence Nightingale, took up the cause of nursing in the Presidency.^10^ Through his initiative, the training of nurse probationers began in 1871 at the General Hospital in Madras. Eventually the Presidencies of Calcutta and Bombay also began to train and employ European, Eurasian and Indian women as nurses in civil hospitals, but an all-India civil nursing service was not formed.^11^

Nursing in the military context had its own distinct origin. In 1857, when the interest in army nursing kindled by Nightingale was still fresh, a major military uprising broke out against British rule. Following this, the governance of British India was transferred from the English East India Company to the Crown in 1858. The political ascendancy of the British empire was heavily dependent upon military strength, which in turn depended considerably on the health of the troops. Among other things, the uprising of 1857 exposed the inadequacies in the medical facilities available to the British army. In response to this, the ‘Royal Sanitary Commission on the Health of the Army in India’ was appointed in 1859. It is particularly significant that Nightingale participated extensively in the inquiries of this Commission.^12^ The Report of the Commission in 1864 brought to the fore the unsatisfactory state of nursing care provided to the British army in India.

A hospital for the European corps had a ‘hospital serjeant’ (sergeant) for maintaining discipline.^13^ Ailing soldiers were often cared for by their ‘comrades’.^14^ However, the bulk of the nursing functions were performed by untrained ‘coolies’ (native attendants). The British soldiers despised the native attendants who were described as ‘inattentive’, ‘lazy’ and ‘apathetic’.^15^ There are references to women performing nursing functions for the army – but they were not professionally trained. Colonial ladies (wives of colonial officials), took up the nursing of wounded soldiers during the uprising of 1857.^16^ Wives and widows of British soldiers, and native women also worked as amateur care-givers in the military hospitals.^17^ The Commission recommended the employment of trained female nurses in the British military hospitals in India, - reflecting the professionalisation and feminisation of nursing underway in Britain.^18^ But this suggestion was not immediately implemented.

## Hesitation towards introducing female nurses in military hospitals

The major reason for the hesitation of the Government of India in introducing female nurses was the financial burden that it would entail. The Controller-General of Military Expenditure prepared an estimate of expenditure required for setting up a complete nursing establishment in India. In 1867, *The Medical Times Gazette* reported:

[C]alculating the cost on the table given by Colonel Broome, the Controller-General of Military Expenditure, the charge to the State for the maintenance of the estimated number of 280 nurses, with three lady superintendents, for the three Presidencies, exclusive of the expenses of outfit and passages of nurses going out from England, and the maintenance of a home depot, would be about £30,000 per annum – a large sum to pay for a doubtful benefit.^19^

Nightingale felt that Colonel Broome’s cost estimate alarmed the Government of India enough to resign from the idea of providing trained female nurses for the army.^20^

Apart from considerations of cost, several other points were raised concerning the introduction of a body of female nurses in the British military hospitals in India. Most of the British officers in India came from a middle class background, or from the upper class landed gentry and aristocracy. On the other hand, British soldiers in India were drawn from the lower rungs of the British society.^21^ While the officers were considered respectable and of good moral stature by virtue of their class status, the soldiers were portrayed as exhibiting bodily intemperance, and unable to regulate their carnal impulses.^22^ Hence, as in Britain, the possibility of soldiers behaving inappropriately with female nurses was expected. But such occurrences in India could affect the image of the British empire as a morally superior and civilised force – an aspect which was touted as one of the many legitimations for colonial rule.^23^ Correspondingly, if women were to attend to inmates affected by venereal diseases (VD), it would be morally abhorrent as VDs were considered as resulting from ‘man’s indulgence in his sexual appetite’.^24^

Considering such concerns in India, Nightingale commented: ‘Nurses among convalescent soldiers in the wards are quite out of place and always will be. They would become playthings and very improper ones’.^25^ Similarly, J. L. Ranking (the Sanitary Commissioner of Madras), while supporting the idea of introducing female nurses to military hospitals, did not want their services to be utilised in either the wards set apart for venereal cases or in the general ward for the treatment of trivial ailments. Rather, ‘it is only in grave cases of disease where strength has to be supported by vigilant administration of restoratives, and by those ministrations that [a] woman only is thoroughly fitted to undertake, that a trained nurse will find her vocation’.^26^ This view was reminiscent of Nightingale’s own vision for female nurses in military hospitals in Britain, and reflective of the trends concerning feminisation of nursing underway there.

Another strand of opinion was that employment of female nurses was unnecessary in the military regimental hospitals, where army men, in peaceful times, were in ‘robust health’ except for ‘slight affections’. Moreover, it was claimed that due to the camaraderie in the army, ailing soldiers would prefer to be tended to by their ‘comrades’ rather than by female nurses with whom they would not have the bond of comradeship.^27^ There was also opposition to the idea of female superintendence over men. Nightingale understood that welcoming female nurses into such a masculine arena of public service as the army was not going to be easy:

[N]ursing in military hospitals requires painful, careful trial, because it must always be an experiment, and a new experiment every time you try it, to put down a few women among a parcel of men, this being the only occupation where a woman is really in actual charge and control of grown-up men.^28^

It is important to note that, contrary to popular belief, the success and recognition received by Nightingale for organising army nursing during the Crimean war, did not immediately guarantee a place for professionally trained female nurses in the army hospitals even in Britain.^29^ By the 1860s, as a result of consistent lobbying by the nursing leadership, women were trained as army nurses at the Royal Victoria Hospital at Netley, and appointed to military general hospitals. They were eventually employed during the Anglo-Zulu war in 1879, which served as an impetus for the formation of the Army Nursing Service by 1881. These developments were also favoured by the reforms in the arena of military health and sanitation in the second half of the nineteenth century.^30^ By 1883, all the military hospitals with more than a hundred beds were required to employ nursing staff who were responsible for the care of the patients and also for the training of orderlies in the wards. A code of regulations for female nursing service of the Army was published in 1884. All the army nurses were required to have undergone training in a civil hospital.^31^ In 1883, the decoration of the Royal Red Cross was instituted to accord public recognition to the British nurses for caring for the soldiers. Such recognition was otherwise denied to women in other arenas of state service. Also, serving the British army in the expanding empire provided a British woman, the greatest opportunity for ‘national service’.^32^ Correspondingly, casting the trained British nurses in an imperial role was seen as a way to boost the public image of the nursing profession following an era of reform and reorganisation.^33^

## The Indian Nursing Service: Organisation and functioning

The developments in Britain were followed by a renewed interest in the introduction of trained female nurses in British military hospitals in India. Lady Roberts (wife of Lord Frederick Roberts, the Commander-in-Chief of British troops in India), was instrumental in lobbying for this cause.^34^ In fruition of the efforts since 1860s, the Secretary of State, in 1887, sanctioned the employment of lady nurses in British military hospitals in India. Thus, was born the Indian Nursing Service (INS). On 21 March 1888, Miss Catharine Grace Loch and Miss Oxley, along with eight other trained English ‘nursing sisters’ arrived in India.^35^ Their induction into the military can be seen, in a way, as another important step in tightening the British hold on India, in terms of its bolstering the health of the army.

The memoir of Catharine Grace Loch serves as one of the important anchors in this paper’s attempt to capture the various dimensions of the organisation of the INS, and the early experiences and challenges of the trained army nurses in India. Loch was born in 1854, into a family of privileged social background. She showed a strong inclination towards nursing, which in her social sphere was still an uncommon vocation for women. At the age of 25, Loch trained to be a nurse at the Royal County Hospital in Winchester. Following this, Loch was appointed as the Night Superintendent at St. Bartholomew’s Hospital in London, where she got nursing experience in the men’s surgical ward. In 1887, Loch was selected to be a Lady Superintendent charged with the task of inaugurating the INS. After more than a decade of service she returned to Britain in 1902, and functioned as a member of the Committee at India Office which dealt with the appointment of nurses to the INS.^36^ Her memoir chronicles the life and work of a professional colonial woman, and her experience of the workings of a predominantly male-run empire. Her story is traced mainly through her official and private letters which provide unusual glimpses into the journey of a pioneering leader of the INS.

The nursing sisters were introduced into British station hospitals in India, which were meant exclusively for the treatment of British men in the army, both officers and soldiers. Their service was not extended to the Indian troops. At the time of their arrival, the hierarchy in a British station hospital was as follows: a medical officer of senior rank had the medical and administrative authority over all functions of the hospital. Other medical officers were in charge of the patients whom they visited generally twice a day. They were also called at other times to attend to emergency cases. Below them was a class of subordinate medical officers designated as assistant-surgeons (or apothecaries), who were responsible for the wards in the absence of the medical officers. They treated the patients according to the orders of the medical officers and were allowed to prescribe treatment in case of emergency. They also supervised the work of untrained orderlies who carried out caregiving activities. The orderlies were assisted by ward ‘coolies’/native attendants who did the menial work in the hospital.^37^

The newly introduced nursing sisters of the INS were required not only to care for the patients, but also to train the orderlies in nursing and to supervise their work. According to the registers of the INS candidates, British women who were single, married or widowed applied to the service, but single women were preferred for appointment.^38^ Candidates had to apply to the Under Secretary of State for India and were required to have at least three years of training and experience in a civil service hospital in Britain.^39^ Nurses trained in India were not preferred as their training was considered inadequate.^40^ The agency of the nursing sisters was characterised by their embodied skill and their embodied expertise.^41^ The sisters acquired these through their training in, and their experience of western medical practice in the civil hospitals in Britain. In India, the sisters were introduced into conditions which challenged their embodied expertise. Firstly, the medical and nursing services, like the army, were itinerant in nature. Hence in military hospitals the doctors, apothecaries, nurses, and orderlies were all frequently transferred, unlike the civil hospitals in Britain where the doctors and nurses worked together for a long time.^42^ The dynamics of work in a military hospital in India was such that mutual trust and confidence could not be cultivated easily. This resulted in a system where the expertise of a nursing sister was not adequately validated or acknowledged. Secondly, western medicine practiced in India was not identical to that practiced in Europe, since it had to adapt according to the conditions and requirements in India. The sisters had to understand and manage the diseases encountered in the ‘tropics’, which had adverse manifestations on European bodies, with their effects intensified by concomitant climatic factors like heat and humidity.^43^ Thus, their embodied expertise had to be reinvented to suit the colonial military medical system.

The lady nurses were an integral part of the military-medical department and could be subjected to court-martial.^44^ There were three grades in the nursing service – ‘nursing sister’, ‘senior nursing sister’ and ‘Lady Superintendent’. The functioning of the INS was spread across four Circles/commands (with headquarters at Rawal Pindi [sic], Meerut, Bangalore, and Poona), each under the charge of a Lady Superintendent. Each Circle had three or four stations.^45^ The promotion of a nursing sister to the position of Lady Superintendent was made by the Chief Medical Officer (CMO) in India on the grounds of ‘experience, administrative capacity, and personal fitness’.^46^ In each station, two to four nurses were employed and one of them (a senior nurse), held the position of Deputy Superintendent. A Deputy Superintendent was supposed to consult the Lady Superintendent on all important matters and submit monthly reports to her. The Lady Superintendent of a Circle had to visit all the station hospitals in her jurisdiction at least once a year and inspect the conditions of work there. She also had to submit a confidential report evaluating the work, capabilities and conduct of each sister to the CMO.^47^ Loch was the Lady Superintendent of the Rawal Pindi command.

Each Lady Superintendent, senior nursing sister and nursing sister received a monthly pay of 300 rupees, 200 rupees and 175 rupees respectively.^48^ The pay was considered quite generous. During a term of service (of five years), a sister was allowed two months of leave on full pay, and sick leave up to a maximum of six months which could be taken in India. After completion of a term, a sister was entitled to one year’s furlough out of India. At the end of each term, she could leave the service or sign an agreement to return for another term. At the end of her full service a sister was given pension based on the duration of her service.^49^

The INS sisters did not experience wartime nursing in India due to reduced military conflict in the colony by the end of nineteenth century. Only rarely did nurses have to be on the move alongside troops during military expeditions. The soldiers who needed treatment for injuries or diseases came to the station hospitals where they were nursed back to health.^50^ The sisters could afford time for leisure and got the opportunity to travel around India. Thus, according to A. Arkle, a senior nursing sister, the INS was overall an exciting prospect for British lady nurses:

Our quarters are always large and comfortable, the pay is good, the amount of leave is most generous, there is a pension at the end of our service, and there is that home-feeling one has in one’s quarters surrounded by one’s little gods. One can keep a pony-trap or bicycle, and one can have one’s live pets about one. This to an animal-lover means a great deal, and I think a real change is good for one. When off duty we can potter around in the garden, play tennis or any other game we like, – golf is a favorite,– and I think a good canter across the country is about the best medicine for a nurse I know of; after it one goes on duty so fresh. I take it that to really remember the men and give them of our best when on duty, we must try to quite forget them when away from the wards.^51^

However, the picture was not entirely rosy. The sisters were placed in charge of special wards in station hospitals which contained the most serious cases. There were healthy months when there were fewer serious diseases and when the work was ‘dull’. Then there were seasons of disease outbreak when the work was ‘wearisome’ which resulted in much exhaustion.^52^ The service of the sisters was crucial in handling epidemiological crises in the army. The nurses dealt with diseases like dysentery, heat apoplexy, rheumatism, liver abscess, pneumonia, malarial and enteric fever, and outbreaks of epidemic diseases like cholera and influenza. There was not much surgical work in the hospitals.^53^ There were instances when nurses fell ill due to the adverse effects of climate, diseases, and work in India. Despite this, there was a regulation that stated that a nurse who took ‘sick leave’ in India would have a third of her pay cut.^54^ Since there were only a few nurses in each station hospital, they worked in three shifts, taking turns on and off duty. Having fewer than three nurses in a hospital was not considered ideal, since it caused the nurses much fatigue especially when faced with circumstances when day and night supervision of patients was required.^55^ Yet, the sisters were often transferred at short notice which disturbed the nursing work in the already minimally staffed station hospitals, leading to patients getting neglected and systematic training of orderlies getting impeded.^56^ These transfers were ordered without consulting the Lady Superintendent– something which invited dismay.

Frustrated at the sudden and unexplained transfer of nursing sisters from one place to another, Loch wrote: ‘It is ridiculous doing things like that, and if I am not to be consulted at all, or even warned about arrangements, what is the good of my existence? I must stand on my own legs, and the first thing is to establish them and to try to show that I have a voice in the matter’.^57^ She also opposed the practice of sisters being arbitrarily ordered by higher authorities, to go to out-stations to nurse individual patients, especially ladies, which was not under the purview of their duties as army nurses.^58^ On one such instance, Loch insisted: ‘I think that just because we are professional we should not be called on to do promiscuous work in addition to our own proper duties’.^59^ It appears that while Loch was anxious to assert her position vis-a-vis her higher authorities as the Lady Superintendent, she was also concerned about the interests of the nursing sisters at large. She was willing to look beyond some of the perceived slights in the larger interest of the profession and of her co-workers. In addressing the several problems faced by the latter, she attempted to solicit favorable solutions from her superiors – which needed a degree of soft-pedalling.

Along with the conditions of work, climate also had adverse effects on the health of the British lady nurses and impacted the chances of their continuous service.^60^ Lady Roberts had anticipated this particular challenge even at the inception of the INS in 1887. She wrote:

Without occasional change to a healthier climate, European ladies could not possibly continue for long to perform the trying and anxious work of nursing in the plains at the most unhealthy seasons of the year. The other alternative is to let the ladies remain in the plains until their health completely breaks down, and then send them to England with more or less chance of recovery. This, besides being cruel to the devoted women who consent to come out for this work, would involve constant change of nurses and an enormous increase of expenditure.^61^

Lady Roberts advocated the provision of ‘homes in the hills’ as health resorts for the nursing sisters in India. But again fiscal conservatism tended to colour the attitude of the colonial government in this regard too: ‘The government of India considers that the money required for this purpose might appropriately be left to the active benevolence of private individuals interested in the welfare of the British soldiers in India’.^62^ Thus, while the colonial government needed the nursing sisters for ensuring the health of its army, it was reluctant to make any expenditure for a scheme to help the health of the nurses serving the army. It was a common practice to have sanatoria particularly in the hills, for the British population especially for the military personnel.^63^ Though the nursing sisters were supposed to be within the military system (in that they could even be court martialed), they were overlooked – in the name of fiscal discipline – when it came to providing them places in the hills to recuperate and rest.

The ‘homes in the hills’ for nurses were set up by private subscription, since the government was not willing to spend for it. ^64^ However, it has to be noted that these ‘homes’ were not something that the nurses themselves welcomed unreservedly. Loch described how expensive houses were bought and furnished at Murree and other places where nurses could be officially sent during leave or in case of sickness. ^65^ She considered this ‘an unfortunate mistake’, though well-intentioned. This was because during their leave the nurses preferred to stay with their acquaintances and friends, rather than being isolated at these ‘homes’. Writing about the home at Murree she noted: ‘…only twice during the two years has a Sister gone there for a week’s sick leave, but if the Home had not existed I have no doubt some other arrangement could have been made’.^66^ The nurses were sent up to the hill station hospitals to work there during the summer months. At Murree, Loch observed that rather than stay in the ‘Home’ which was far away from their place of work, it was more convenient for the sisters on duty to have their accommodation at the quarters close to the hospital.^67^ Hence Loch saw the ‘homes in the hills’ as unnecessary.

Loch rather insisted upon better conditions of work and stay for the nurses. She preferred an increase in the number of nurses in the service, in order to afford rest to the nurses, and thereby enhance their efficiency. In 1896 there were only fifty-two nursing sisters employed in the entire service.^68^ Requests for increasing the number of nurses were frequently made, but any positive action was delayed by the government because it was considered expensive. Disappointed with such a state of affairs, Loch wrote: ‘Yet after all the palaver and parade we cannot get even what we want for our patients, and we are considered unreasonable and exacting when we ask for things, and get told that if we make ourselves expensive, we must not expect Government to increase our numbers’.^69^ In 1901, it was proposed to provide an increment of twenty-five rupees to the monthly salary of those nursing sisters and senior nursing sisters who had completed five years of service. This proposal was ‘negatived’ by the Government of India on the ground that the pay and allowances granted to the lady nurses in the INS were already considerably high compared to those given in Britain.^70^

From the instances noted above, the attitude of the colonial government towards the nursing service can be understood. Firstly, as a newly instituted service, the colonial authorities maintained a tight grip on its functioning. This brought them in conflict with the Lady Superintendent of the service, who was accorded a semblance of power - but with no real authority. The suggestions and opinions of the Lady Superintendent were often ignored or simply not sought. Secondly, the colonial government was reluctant to spend towards the service which was considered as an experimental attempt. The salaries of the sisters remained static, and the quality and welfare of the service was compromised as sufficient numbers were not recruited due to the financial stringency of the colonial government.

## Nursing sisters in a male dominated work-place

Before the introduction of the nursing sisters to the military hospitals, many of the orderlies, assistant-surgeons, and medical officers (who were all men), had little or no experience of working with a lady nurse.^71^ The presence of female agency in a male-dominated arena of work was bound to create tensions. At the British station hospitals, the sisters were placed in charge of supervising the nursing work of male orderlies drawn from the various local British combatant units. They also were given the responsibility of imparting nursing knowledge to the orderlies and training them for medical work. These nursing orderlies were described as ‘totally ignorant’ of the most rudimentary principles of nursing. Hence the sisters were required to instruct them in every practical and basic detail related to nursing, and by ‘force of example’ help the orderlies to develop interest in nursing duties.^72^ This can be counted among the few scenarios in the nineteenth and in the early-twentieth centuries where women were in charge of instructing adult men.

A system of training nursing orderlies evolved in which military men could volunteer to take a course which included lectures by medical officers on first aid and elementary nursing, in addition to a ‘stretcher drill’ course. On satisfactory completion of the course, the trainees were sent, generally for two months, into wards for practical learning under the sisters, after which they received a nursing certificate.^73^ The sisters were required to train about ten to twenty probationing orderlies at once, in that very short time, which they found difficult.^74^ Loch was concerned about the ‘danger to the system’ whereby, for many people a ‘certificated orderly’ conveyed the idea of a ‘trained orderly’, which he certainly was not to begin with.^75^ From her observation, the real gain of knowledge and practical habit formation as nursing orderlies happened only when they joined the job after the so-called training.

The possession of a nursing certificate entitled an orderly to earn an extra pay of four annas per day for hospital work. This gave official recognition to the nursing orderlies, and incentive to learn and perform nursing work.^76^ For each ward with an average of twenty-five beds, two orderlies were assigned. Each orderly worked two alternate shifts of six hours each. The opinion of the sisters regarding the orderlies was ambivalent, and swung between appreciation and complaint. Arkle (a senior nursing sister), wrote: ‘I find the orderlies much better and more willing to learn than I expected. I have seen them so infinitely gentle when handling a sick comrade’.^77^ This was not always the case. The sisters did face difficulties in managing the orderlies. The supply of orderlies changed frequently with the coming and going of regiments at a station. This was a huge disadvantage to the sisters as they often lost the orderlies whom they had acquainted with hospital work, only to receive a new set of inexperienced ones. The orderlies were also often found to be drunk or sleeping, instead of taking care of the patients in the ward, which required the sisters to take on the responsibility themselves.^78^ Loch thus recorded her experience with orderlies at Rawal Pindi in one of her letters:

For one thing we have been going through much tribulation with orderlies. We have some very good men in our original ward, but in the new ward and for the extra cases in the verandah we had a wretched lot belonging to a regiment which was only passing through Pindi. They did not care a fig, and were quite stupid and not civil and frequently drunk. Several times I had to put the night orderly to bed to get rid of him and do his work myself.^79^

While the doctors and assistant surgeons had ranks in the military medical system, the nursing sisters were not given any. ^80^ In the absence of a nominal rank in relation to the orderlies, the sisters often faced instances of insolence and disobedience.

There was a sense of competition in the interaction between the assistant surgeons/apothecaries and the nurses. Loch noted that the assistant surgeons were inclined to view the sisters with ‘very jealous eyes’.^81^ This was probably because after the introduction of the nursing sisters, the supervision of nursing work, which was earlier under the purview of the assistant surgeons, had become the responsibility of the sisters. There was a lack of clarity about where the sub-medical charge of an assistant surgeon ended and where the nursing charge of a sister began. Hence the sisters felt that the assistant surgeons interfered with their work and encroached upon their sphere of responsibility.

The inadequate delineation of responsibility in the military medical system, and the refusal of the government to grant a nominal rank to the sisters undermined the authority and position of the sisters in relation to the orderlies and the assistant surgeons. Loch, in her position as Lady Superintendent, was not satisfied with mere praise for the work of the nursing sisters, without actual legitimation by the government. She wrote:

I feel sure that in this horrid country Nursing Sisters never will get a proper recognized position in spite of all the palaver of the military authorities. Of course, we are everything that is delightful and revered and much thought of in conversation, but when it comes to the point, we must be ministering angels only without a definite responsibility or position of any sort or kind, or any recognized status with regard to soldiers, orderlies or apothecaries (members of the Subordinate Medical Department), and naturally they take advantage of it, seeing that everyone else’s rank is fixed to a hair’s breadth.^82^

In a hierarchical system dominated by men, the issue of granting a recognized status to female agency was ignored. Quite exasperated with the government on this matter, Loch pondered: ‘Why must it be always thought that in connexion [sic] with women things may be left vague and unbusinesslike?’.^83^ However, the nursing sisters did not end up entirely as passive agents. With all the limitations and challenges, they yet constantly sought to define for themselves their place and role in the healthcare structure.

The medical officers tended to treat the nursing sisters as if they were probationers or ‘supernumeraries’, and showed more deference towards the assistant surgeons. The advice of the sisters against unsuccessful methods of treating patients, was often disregarded by the inexperienced young medical officers. In general, Loch maintained a cordial professional relationship with medical officers. According to her, ‘In the abstract, the doctor must always have the authority and the nurse must obey, but when one has worked many years, one often knows best all the same’.^84^ But the medical officers tended to behave in an ‘autocratic’ manner towards the sisters. Loch narrates one such instance:

A certain convalescent was ordered by the doctor a dose of castor oil and had it; for some unexplained reason the dose upset the man utterly, started violent irritation and diarrhoea, and the poor fellow, who had been practically well, was getting awfully bad when the Sister on duty gave him a dose of stock astringent, an excellent one always kept ready in the hospital, and in an hour he was all right. But the doctor got furious and declared that we had no business ‘to prescribe’ for patients, and that if so he could not be responsible for them and so on, and he removed every single stock bottle and store thing in our cupboards, so now we have nothing at hand.^85^

Thus, it can be understood that a nurse was not supposed to administer any medicine or treatment to the patient unless prescribed by the medical officer himself. This rule was to be followed even in emergency conditions when the patient was likely to collapse and when there were no medical officers or apothecaries available to prescribe treatment.^86^ In case of any conflict with medical officers, the nurses often felt unsupported because the head of administration, being a doctor, would back the doctors. Hence as female agents, the nursing sisters faced disheartening instances when their sense of judgment was not trusted, and the expertise they possessed through experience was not given due credit.

## Scrutiny and appreciation: Lady nurses as agents of the empire

The sisters of the INS, the pioneering all-women’s service within the army, were under scrutiny not only in their workplace, but also socially. A high standard of conduct and work was expected out of every individual nurse, since their actions could retard or advance the cause of nursing in India ^87^:

[E]very individual Nurse who earnestly cares for her profession may feel that she is one of a small band of pioneers in a new sphere, where skilled nursing has hitherto been extraordinarily unknown; that she is watched with interest, both by the public at large and by the Government she is serving; and that the success and encouragement given to trained nursing in India may be immensely forwarded by her individual efforts, which will actually help to bring a real improvement in the nursing of the military hospitals generally a step nearer realization.^88^

Negative opinions regarding the whole scheme of nursing service were circulated from time to time in the papers, accusing the nurses of disobedience of orders and mismanagement, which affected the morale of the nurses.^89^ Loch wrote: ‘…they keep on writing nasty things in the newspapers. But it is not a bit of good really minding, though it is disheartening, when one knows one works fifteen times harder than anybody else, to be abused in addition’.^90^

A military station was a censorious environment for the British nursing sisters who were mostly young unmarried women, devoid of familial regulations in a faraway land.^91^ Loch warned the sisters not to be overly excited by their supposedly independent position. She advised them to live ‘quietly and unostentatiously’, and refrain from ‘parading’ their independence.^92^ On closer look, it can be understood that the nurses’ being away, in fact, reduced their independence of action and increased their vulnerability and liability to be judged unfairly:

But the fact that they are young women, living without any protection from relations or friends, renders their position in some ways a difficult one. Instead of being more independent, they have practically less safe liberty of action than many a girl living in her father’s house might safely enjoy. Nurses out here are far more prominent in the eyes of the community than Nurses in England. Everyone criticizes them; and should one of them, perhaps from mere thoughtlessness, ‘get talked about,’ as the saying is, there is no one to stand up for her or to vouch for her in any way. Sometimes they are extravagantly admired for the work they do, but a large number of people are always to be found who are only too ready to find fault.^93^

The insistence on more probity, conformity, and professional identity within the service was signaled by the introduction of uniform (duty dress) in 1900. The sisters were rendered easily noticeable by their uniform which they were required to wear ‘at all times’, unless they were on leave or attending evening entertainments.^94^ The sisters got invited to station gaieties (like dances), arranged for the officers and their families. However, the government discouraged them from engaging extensively in such activities since it invited gossip and criticism against them. They could be accused of being more interested in these pleasures than in the service of patients. Moreover, such amusements were seen as an interruption to the routine, and something which weakened their interest and commitment towards work.^95^ Hence, to retain the image of the INS, even the personal activities and desires of the sisters were regulated.^96^ The sisters were advised to exercise ‘caution’ and ‘self-restraint’ in both professional and social circles.

At the time of appointment, a nursing sister was required to be over twenty-five and under thirty-five years of age.^97^ There was a preference for candidates considered to be ‘ladies’ of unquestionable social position. The candidates applying for appointment in INS were specifically required to produce ‘Original letters of personal recommendation, including one from a lady of position in Society, who must state that the candidate is a fit, and in every way, desirable person to enter a service composed of ladies of good social position with whom she will associate’.^98^ Thus, the colonial authorities wanted to confirm the social standing of the nursing sisters, before they could be considered for service in India.

All of this has to be seen in the backdrop of very significant changes in Britain with regard to the social standing of nurses as professionals, which had clear class dimensions too. Prior to the reforms in the field of nursing in the mid-nineteenth century in Britain, nursing as an occupation was only taken up by working-class women, while nursing for the sick in the domestic sphere had been the duty of women in all social classes. It was not considered respectable for women of good social standing to take up nursing as an occupation, as nurses were cast as dirty, drunken and sexually promiscuous women. However, due to the reforms initiated in the mid-nineteenth century, nursing was transformed into an occupation requiring rigorous discipline and organised training. These developments improved the image of nursing and attracted middle-class ‘ladies’ into the occupation, apart from working class women.^99^ The participation of ‘ladies’ who were represented as being immune to sexual temptation, especially from lower-class patients, in turn reciprocally helped in elevating the image of nursing to be a respectable occupation.^100^

Apart from providing nursing care to both the British officers and soldiers, the sisters were additionally placed in a position of authority over the soldiers, whom they were to supervise and train as nursing orderlies. Under this arrangement, the sisters were expected to be of a superior social standing than the soldiers. It was opined that if sisters were of the same class as the soldiers, they would not be respected or obeyed.^101^ Loch preferred ‘lady’ nurses, and was not in favor of nurses from the lower-classes being appointed into the INS. She asserted:

I do think it will be a grave mistake not to send out ladies. First of all, it is hard on those who are not, because naturally they are sniffed at and make no friends; next, it is hard on those who are, because people always charitably judge the many by the few, and they will find themselves thrown out of their proper position in life on account of their colleagues.^102^

Hence appointing lower class British women as nursing sisters was considered detrimental to the discipline, unity and quality of the service, which could have adverse effects on the image of INS as a prestigious colonial service.

At this point, it would be useful to delve briefly into the significance of the presence of British ladies in colonial India. During the nineteenth century, the British Victorian middle-class ‘ladies’ in India served a performative function as representatives of the ‘civilised’ culture of the British colonisers.^103^ Though British women in India were integral to what constituted the colonial image, Sara Suleri opines that it was a long-standing tradition for them to be on the periphery of colonisation, as they were rarely required to handle any responsibility outside the domestic sphere. Hence, they remained fairly immune to the workings of British imperialism.^104^

In the course of the nineteenth century, an arena for ‘women’s work for women’ emerged in colonial India, initially within the realm of missionary activities (both British and American), and later in the field of colonial medicine as well, which focused on providing medical relief for Indian women.^105^ This proved to be a favorable avenue especially for educated, middle class British ladies to transition into the public sphere for socially accepted, paid work.^106^ These British women associated their work with the idea of the ‘civilising mission’, and played an ideational role in extending the influence of the empire to the ‘natives’.

In contrast, the British ladies employed as army nurses in the INS, were distinctly involved in ‘women’s work for men’, performing a feminised role in a masculine public arena. However, their interaction with the natives was limited. Thus, the nursing sisters add plurality to the trope of the ‘British lady’ in India. Sir James Crichton-Browne described the sisters as ‘handmaidens of humanity’ for their dedicated service to ailing soldiers, while also calling them ‘militants’ fighting against the relentless attack of diseases that affected the soldiers.^107^ Loch highlighted the contribution of nurses stating that ‘…many a young soldier, exiled far from home and friends, will owe his life directly to her skill and care’.^108^ These comments position the British lady nurses as agents of the empire, who played an instrumental role in maintaining the vitality of the army, which was a prominent axis of colonial power. Thus, unlike the earlier British women on the periphery of colonial activity, the British lady nurses, through their medical engagement with the military, became a more integral element of the colonial machinery.

## Progressive developments in the nursing service

By the beginning of the twentieth century, the significance of the army nursing services in both Britain and its colonies came to be recognised. In 1901, the Brodrick’s Committee for the reorganisation of army medical services in Britain recommended the amalgamation of the Army Nursing Service (in Britain) and the INS into the Queen Alexandra’s Imperial Military Nursing Service (QAIMNS).^109^ This proposal was rejected by the Indian authorities. In 1903, the INS was just renamed the Queen Alexandra’s Military Nursing Service for India (QAMNSI).^110^ One among the four Lady Superintendents was given the new designation of Chief Lady Superintendent.^111^ (Catharine Grace Loch was the first Chief Lady Superintendent of the QAMNSI).^112^ She was the recognised advisor to the Director of Indian Medical Service in matters concerning ‘interior economy and welfare’ of the nursing service.^113^ In 1905, there was a suggestion to appoint a Matron-in-Chief for the QAMNSI. This was rejected on the grounds that owing to the size of India, it was more efficient to maintain the four Lady Superintendents who could provide advice on all matters relating to their charges.^114^

In 1902, the Government of India had proposed a plan for the augmentation of the strength of the nursing service by seven senior nursing-sisters and thirty-two nursing sisters in the course of three years.^115^ This plan was gradually implemented with considerable success, and by 1906, the total strength of the establishment was eighty-four, with four Lady Superintendents, fifteen senior nursing sisters and sixty-five nursing sisters.^116^ The lady nurses worked with hardly any chance of promotion, and some of them worked for more than ten years without obtaining an increment in pay. In 1907, the Government of India sanctioned an increment of twenty-five rupees to the monthly salary of those nursing sisters and senior nursing sisters with over five years of service. At that time there were sixteen senior nurses and seventeen nursing sisters eligible for this increment, the total cost of which was estimated to be 9,900 rupees per annum.^117^ These developments point to the reality that the nursing service had become firmly established in the military hospitals in India.

Another significant development was that, by the beginning of the twentieth century, the gendering of the nursing profession as feminine was reaffirmed, and army nursing by women came to be more readily accepted:

Apart from special training, patience, sympathy, gentleness, and devotion are most essential qualities required for successful nursing, and in these respects women excel men. Nursing is a woman’s special sphere. If it takes time to make a soldier it takes much longer to make a really good nurse, the value of whose services when obtained, is however, incalculable.^118^

It is interesting to note that the male nursing orderlies, under the supervision of sisters, were advised to observe obedience, truthfulness, cleanliness, temperance, patience, kindness, gentleness and quietness.^119^ These qualities, often portrayed as desirable feminine traits, were being expected from male orderlies, but devoid of its gendered associations. Hence it can be assumed that these qualities were, by then, integrated as the core values of nursing practice itself.

An important marker for the progressive professionalisation of the nursing service in India, was the strict insistence on three years of training in nursing. In 1902, the candidates were required to state where and for how long they had undergone training. They were required to produce ‘Original Certificates as to efficiency in Medical and Surgical Nursing from the Medical Officers and from the Lady Superintendent or Matron of the Hospital where the training has been undergone’.^120^ By virtue of their training, the nursing sisters, embedded within the western structure of medical knowledge, served as intermediaries between the ‘expert’ medical officer and the ‘amateur’ orderlies who were required to do practical medical activities.

In 1912, Mary R. Truman, a senior nursing sister from the QAMNSI, published her *Simple Lectures on Nursing for Soldiers in India,* as a ‘record of personal practical experiences’ written for soldiers being trained as nursing orderlies. In its Preface she wrote:

This book is written in the hope that it may prove useful to the various classes of soldiers in India, who annually undergo a course of training in nursing duties to enable them to nurse their sick comrades. It does not aim at being a complete treatise in nursing, but I have tried to include in concise form and in plain language some of the most important facts that have helped me, in the hope that they may help others.^121^

Truman provided instructions for nursing several ailments and diseases including wounds and fractures. She explained the meaning of medical terms, the functions of several medical equipments, the modes of administration of medicines, the modes of spread of infectious diseases and their symptoms. Various basic aspects of sick-nursing like maintaining cleanliness, bathing, dressing and nutrition of patients were also explained.^122^ The book is a testament to the role of a nursing sister in codifying nursing practice in military hospitals in India, and the effort to build a pedagogic tradition for the nursing orderlies.^123^ This was also a result of the nursing sisters’ growing confidence in their expertise, developed through their experiences in India. The expertise of the QAMNSI nurses was acknowledged when in 1916 they were sent to nurse the troops from British India, who were fighting the Mesopotamian Campaign of the First World War.^124^

## Shortcomings of nursing service for the army

Despite these developments, the nursing service in the army was noted as ‘far from satisfactory’ in the *Report of the Committee Appointed by the Government of India to Examine the Question of the Reorganization of the Medical Services in India* in 1920. Several factors impeded the quality of nursing in the British military hospitals in India. There was no marked increase in the strength of the nursing service. The establishment consisted of only ninety-one members including four Lady Superintendents, sixteen senior nursing sisters and seventy-one nursing sisters in 1920.^125^ The nurses had very limited chances for promotion, and some of them retained the same grade as they had when they joined, even up to their third term. The senior nurses often had no official authority over their juniors by virtue of their experience. A Lady Superintendent had hospital duty alongside her administrative responsibilities, making it difficult for her to exercise disciplinary functions over the members of the service. Thus, the nursing administration suffered from lack of a well-defined ‘chain of responsibility’. There was also a level of ‘professional deterioration’ caused by the want of facilities to keep the nurses updated with ‘modern knowledge and methods’.^126^ This was in some ways similar to other areas of colonial knowledge practice whereby, for instance, the practitioners of science in the ‘periphery’ felt deficient and deprived of some of the advantages of the ‘metropolis’ - especially the opportunity of being acquainted with the latest developments in such centers in Europe.^127^

A deficiency in discipline was also observed in the work of the nursing orderlies. A soldier was not assigned nursing duty as orderly for more than six months, since a prolonged period in the hospital was regarded as detrimental to his efficiency as a ‘fighting soldier’. Though the orderlies were provided training, it was described to be of a ‘perfunctory’ nature, because of the limited time in which it was conducted. They were ‘at the will’ of the commanding officers who could call them out of their nursing assignment for regimental training or into mobilization.^128^ Thus, the trained nursing sisters seem to have had no real authority over the male nursing orderlies.

Apart from partial training of orderlies and frequent change of personnel, the quality of nursing in the British station hospitals was also believed to be affected by the employment of Indian ward servants to perform nursing duties – something that was explicitly considered to be a ‘mistake’. Such servants were described as men of ‘very low caste’ who were ‘venal and dirty’ in their habits, and were portrayed as indifferent and untrustworthy when it came to performing even the most menial nursing tasks.^129^ Thus the natives were considered unfit to provide western nursing care. They were not given any nursing training like the British nursing orderlies got.

All this has to be contrasted with the scenario in Indian station hospitals meant for Indian soldiers, and the station family hospitals meant for the families (women and children), of British officers and soldiers. In the Indian station hospitals ‘scientific nursing’ was pronounced ‘non-existent’ since there were no trained nurses employed there. Untrained Indian sepoys were attached to the hospital as ward orderlies. These men were as a rule those who had failed in some ways as soldiers.^130^ In the station family hospitals, the nursing functions were the responsibility of a matron who was trained and certified in midwifery. They were also assisted by some ‘menial female servants’. Unlike the nursing sisters in the station hospitals for British army men, the matron in a station family hospital had no training in general nursing. Regarding the matron, it was reported: ‘…though she may be an efficient midwife, she cannot have that knowledge of ordinary sick-nursing which is essential for the proper attendance on the various classes of cases that find admission to the hospitals’.^131^ The lack of nursing sisters in Indian station hospitals and station family hospitals further points to the reality that the government was willing to employ trained nurses only for the British men in the army.

## Conclusion

The nursing service for the army provided an important arena for the unfolding of female agency in the British empire. The introduction of trained female nurses in colonial India was initially resisted on considerations of cost, and over concerns regarding the moral implication of feminine presence in a masculine military environment. These hesitations were gradually overcome when, as a result of reforms in Britain, nursing work began to be recognised as a feminised profession. These developments coupled with effective lobbying led to the formation of the Indian Nursing Service, through which British nursing sisters were employed in station hospitals meant exclusively for British soldiers and officers.

As a newly instituted service, the patriarchal colonial government maintained a tight hold over its functioning, often repudiating the opinions of the Lady Superintendent in charge of the service - who was granted a semblance of power, but no real authority. The government’s financial stringency impeded the expansion of the service, and compromised the welfare of the sisters. Moreover, the ambiguous framing of the role and status of female nurses in an otherwise rigid, male dominated military medical hierarchy, created tensions in the relationship of the sisters with the male nursing orderlies, sub-medical and medical officers, and undermined the nurses’ agency. Thus, in the initial years of the INS, the government employed female agency without legitimising it.

The agency of the nursing sisters which was characterised by their embodied skill and expertise, acquired through their training and experience in civil hospitals in Britain, was challenged in the colonial setting. The expertise of the sisters had to be adapted to handle the epidemiological conditions in India. Moreover, the itinerant nature of the military, medical, and nursing services in India, along with gender-based tensions, were detrimental for cultivating an environment of mutual trust and confidence between the medical and nursing staff in military hospitals. Due to this the expertise of the sisters was not adequately validated. Despite these challenges, the sisters were instrumental in setting army nursing on a professional footing, by using their expertise for codifying nursing practices for military hospitals, and building a pedagogic tradition for training nursing orderlies.

The nursing sisters as part of a pioneering, all-women service, were held to scrutiny and criticism both professionally and socially. The sisters, who were mostly young single women, were expected to uphold the dignity of their professional identity even while engaging in the social life in military stations. For this purpose, it was insisted that women of ‘good’ social background be appointed to the service, so that they could maintain the ideals of feminine purity while engaging in a masculine military setting, and elicit deference from the nursing orderlies who were British soldiers of ‘inferior’ social class. Thus, in order to maintain the image of the service, the colonial authorities imposed conditions and regulations on the sisters, which reflected the prevalent gender and caste notions.

By the beginning of the twentieth century, the nursing service for the army was firmly established in colonial India. The role of the British lady nurses in maintaining the health of the army, made them an integral part of the colonial machinery. However, the government continually showed an attitude of neglect towards the nursing service and hesitated to spend in that domain. There were no trained nurses employed in the hospitals for the Indian troops. This left the nursing service in the military hospitals with much to be desired even three decades after its inception. However, one cannot deny its foundational role in an important aspect of health care, as also its significant place (however small) in the broader imperial order.

## Footnotes

1 A select and broadly representative sample includes: David Arnold , Colonizing the Body: State Medicine and Epidemic Disease in Nineteenth-Century India (Berkeley: University of California Press, 1993); Mark Harrison , Public Health in British India: Anglo-Indian Preventive Medicine 1859-1914 (Cambridge: Cambridge University Press, 1994); Biswamoy Pati and Mark Harrison (eds), Health, Medicine and Empire: Perspectives on Colonial India (New Delhi: Orient Longman, 2001); Biswamoy Pati and Mark Harrison (eds), The Social History of Health and Medicine in Colonial India (London: Routledge, 2009); Sandhya Polu , Infectious Disease in India, 1892-1940: Policy-Making and the Perception of Risk (Basingstoke: Palgrave Macmillan, 2012); Deepak Kumar & Raj Sekhar Basu (eds), Medical Encounters in British India (Delhi: Oxford University Press, 2013); Pratik Chakrabarti , Medicine and Empire, 1600-1960 (Basingstoke: Palgrave Macmillan, 2014); Radhika Ramasubban,‘Imperial Health in British India, 1857–1900’, in Roy Macleod and Milton Lewis (eds), *Disease, Medicine, and Empire: Perspectives on Western Medicine and the Experience of European Expansion* (London: Routledge, 1988), 38–60; Anil Kumar , Medicine and the Raj: British Medical Policy in India, 1835-1911 (New Delhi: Altamira Press, 1998); Sunil S. Amrith , Decolonizing International Health: India and Southeast Asia, 1930-65 (Basingstoke: Palgrave Macmillan, 2006); Projit Bihari Mukharji , Nationalizing The Body: The Medical Market, Print and Daktari Medicine (London: Anthem Press, 2009); Poonam Bala , Contesting Colonial Authority: Medicine and Indigenous Responses in Nineteenth and Twentieth-Century India (Plymouth: Lexington Books, 2012); Madhuri Sharma , Indigenous and Western Medicine in Colonial India (New Delhi: Foundation Books, 2012); Sujata Mukherjee , Gender, Medicine, and Society in Colonial India: Women’s Health Care in Nineteenth and Early Twentieth-Century Bengal (New Delhi: Oxford University Press, 2017).

2 These works mainly discuss the various aspects of civil nursing in colonial and post-colonial India. See Madelaine Healey , Indian Sisters: A History of Nursing and the State, 1907-2007 (New Delhi: Routledge, 2013); Sujani K. Reddy , Nursing & Empire: Gendered Labor and Migration from India to the United States (Chapel Hill: The University of North Carolina Press, 2015); Meera Abraham, *Religion, Caste, and Gender: Missionaries and Nursing History in South India* (Bangalore: B.I. Publications, 1996); Panchali Ray, *Politics of Precarity: Gendered Subjects and the Health Care Industry in Contemporary Kolkata* (New Delhi: Oxford University Press, 2019).

3 See Deborah Gorham , The Victorian Girl and the Feminine Ideal, Routledge Library Editions: Women’s History, Vol. 19 (London, New York: Routledge, 2014).

4 See Carol Helmstadter and Judith Godden , Nursing Before Nightingale, 1815 - 1899 (Abingdon: Routledge, 2016).

5

Sandra Holton , ‘Feminine Authority and Social Order: Florence Nightingale’s Conception of Nursing and Health Care’, Social Analysis: The International Journal of Anthropology, 15 (1984), 59–72 : 61–63.

6 See Christopher J. Gill and Gillian C. Gill , ‘Nightingale in Scutari: Her Legacy Reexamined’, Clinical Infectious Diseases, 40, 12 (2005), 1799–1805 1590926910.1086/430380; Carol Helmstadter, ‘Class, Gender and Professional Expertise: British Military Nursing in the Crimean War’, in Jane Brooks and Christine E. Hallett (eds), *One Hundred Years of Wartime Nursing Practices, 1854-1953* (Manchester: Manchester University Press, 2015), 23–41.

7 Holton, *op. cit.* (note 5), 60.

8
*Subsidiary Notes as to the Introduction of Female Nursing into Military Hospitals in Peace and in War* (London: Harrison and Sons, 1858), 1–133: 15.

9 See Healey, *op. cit.* (note 2).

10 Gérard Vallée (ed.), *Florence Nightingale on Health in India*, The Collected Works of Florence Nightingale, Vol. 9 (Ontario: Wilfrid Laurier University Press, 2006), 950–955.

11 See Healey, *op. cit.* (note 2).

12

Jharna Gourlay , Florence Nightingale and the Health of the Raj (Aldershot: Ashgate, 2003), 28 .

13
*Report of the Commissioners Appointed to Inquire into the Sanitary State of the Army in India; With Abstract of Evidence, and of Reports Received from Indian Military Stations* (London: Her Majesty’s Stationery Office, 1864), 330.

14
*Reports of the Royal Commission on the Sanitary State of the Army in India.* Vol. II (London: Her Majesty’s Stationery Office, 1863), 38.

15
*Report of the Commissioners, op. cit.* (note 13).

16 Sam Goodman, ‘Lady Amateurs and Gentleman Professionals: Emergency Nursing in the Indian Mutiny’, in Helen Sweet and Sue Hawkins (eds), *Colonial Caring: A History of Colonial and Post-Colonial Nursing* (Manchester: Manchester University Press, 2015), 18–40: 19.

17 Vallée, *op. cit.* (note 10), 947; *Reports of the Royal Commission*, *op. cit.* (note 14), 38.

18 Vallée, *op. cit.* (note 10), 953.

19 ‘Female Nurses in Military Hospitals’, *Medical Times and Gazettes: A Journal of Medical Science, Literature, Criticism, and News,* 2, July to December (1867), 230–231: 231.

20 Vallée*, op. cit.* (note 10), 969.

21

P. E. Razzell , ‘Social Origins of Officers in the Indian and British Home Army: 1758-1962’, The British Journal of Sociology, 14, 3 (1963), 248–260: 248.

22 These differential characterisations of the British officers and soldiers in India was a reflection of the class notions existing in Britain at that time. See Erica Wald, ‘Health, Discipline and Appropriate Behaviour: The Body of the Soldier and Space of the Cantonment’, *Modern Asian Studies,* 46, 4 (2012), 815–856: 851; Douglas M. Peers, ‘Privates off Parade: Regimenting Sexuality in the Nineteenth-Century Indian Empire’, *The International History Review*, 20, 4 (1998), 823–854: 836.

23 Waltraud Ernst argues that it was the British anxiety to preserve their image as a superior people that caused them to hide away and send home British lunatics in India, who could tarnish and bring disrepute to colonial rule. See Waltraud Ernst , Mad Tales from the Raj: The European Insane in British India, 1800-1858 (London: Routledge, 1991), 126 . Also see Ann Laura Stoler , ‘Making Empire Respectable: The Politics of Race and Sexual Morality in 20th-Century Colonial Cultures’, American Ethnologist 16, 4 (1989), 634–660.

24 ‘Female Nurses’, *op. cit.* (note 19).

25 Vallée, *op. cit.* (note 10), 965.

26

J. L. Ranking , Report on Military Sanitation in the Presidency of Madras (Madras: Government Press, 1869), 18 .

27 ‘Female Nurses’, *op. cit.* (note 19), 230–231.

28 Vallée, *op. cit.* (note 10), 965–966.

29

Anne Summers , Angels and Citizens: British Women as Military Nurses, 1854-1914 (London: Routledge & Kegan Paul, 1988), 2 .

30 See Juliet Piggott , Queen Alexandra’s Royal Army Nursing Corps (London: Cooper, 1975).

31 F. A. E. Crew (ed.), *The Army Medical Services.* Vol. 2, History of the Second World War, United Kingdom Medical Series (London: Her Majesty’s Stationery Office, 1955), 2.

32 Summers, *op. cit.* (note 29), 6.

33 Rosemary Fitzgerald, ‘Making and Moulding the Nursing of the Indian Empire: Recasting Nurses in Colonial India’, in Avril A. Powell and Siobhan Lambert-Hurley (eds), *Rhetoric and Reality: Gender and the Colonial Experience in South Asia* (New Delhi: Oxford University Press, 2006), 185–222: 186.

34 ‘Lady Robert’s Proposal of Trained Nurses for India’, *The British Medical Journal,* October (1887), 744.

35 Sarah A. Tooley, *The History of Nursing in the British Empire* (London: S. H. Bousfield & Co, 1906), 349.

36

Catharine Grace Loch , *Catharine Grace Loch, Royal Red Cross, Senior Lady Superintendent*, Queen Alexandra’s Military Nursing Service for India: A Memoir, A. F. Bradshaw (ed.) (London: Henry Frowde, 1905), 1–6 .

37
*Ibid.*, 338–339.

38 ‘Specimens of Forms Used in Recruitment of Indian Medical Service Officers and Nurses of Queen Alexandras’s Military Nursing Service for India’, 1897-1911, IOR/L/MIL/7/19351, British Library; The nursing sisters were likely to leave the service if they were married. See ‘Indian Nursing Service: Registers of Indian Nursing Service Candidates, 1887-1902, IOR/L/MIL/9/430, British Library.

39

A. Arkle , ‘The Indian Army Nursing Service’, The American Journal of Nursing, 2, 9 (1902), 652–655: 652.

40 ‘Indian Nursing Service: Registers of Indian Nursing Service Candidates, 1903-1916, IOR/L/MIL/9/431, British Library, 49–50.

41 For more understanding on ‘embodied skill’ and ‘embodied expertise’, see Paola Bertucci , ‘Spinners’ Hands, Imperial Minds: Migrant Labor, Embodied Expertise, and the Failed Transfer of Silk Technology across the Atlantic’, Technology and Culture, 62, 4 (2021), 1003–10313469015710.1353/tech.2021.0152.

42 Loch, *op. cit.* (note 36), 344.

43 See Harrison, *op. cit.* (note 1).

44 Arkle, *op. cit.* (note 39).

45 Loch, *op. cit.* (note 36), 57.

46 Arkle, *op. cit.* (note 39).

47 Loch, *op. cit.* (note 36), 338.

48 ‘Grant of an Increase of Pay to Each Nursing Sister and Senior Nursing Sister After Completing Five Years Service in Either of these Grades’, Government of India (Army Department), Principal Medical Officer’s Division, Medical Department – Nurses-A, Proceedings, July 1907, Nos. 975–981. National Archives of India (hereafter NAI).

49 Arkle, *op. cit.* (note 39), 653–655.

50 Loch, *op. cit.* (note 36), 347.

51 Arkle, *op. cit.* (note 39), 655.

52 Loch, *op. cit.* (note 36), 326.

53
*Ibid.*, 347.

54
*Ibid.*, 70.

55
*Ibid.*, 57-58.

56
*Ibid.*, 37-39.

57
*Ibid.*, 69.

58
*Ibid.,*37–39.

59
*Ibid.*, 40.

60

Verney Lovett , Report of the Committee Appointed by the Government of India to Examine the Question of the Reorganization of the Medical Services in India (London: His Majesty’s Stationery Office, 1920), 53 .

61 ‘Hill Homes for Nursing Sisters in India’, *The Indian Magazine*, January-December (1887), 509.

62
*Ibid.*

63 See Dane Keith Kennedy , The Magic Mountains: Hill Stations and the British Raj (Los Angeles: University of California Press, 1996); Hyde Clarke , ‘The English Stations in the Hill Regions of India: Their Value and Importance, with Some Statistics of Their Products and Trade’, Journal of the Statistical Society of London, 44, 3 (1881), 528–573: 560 .

64 Lady Roberts’ Fund was started with the objective of providing homes in the hills for nurses working in military hospitals in India. See Tooley, *op. cit.* (note 35), 350.

65 Murree was a hill station in the Punjab region of British India.

66 Loch, *op. cit.* (note 36), 45.

67
*Ibid.*

68 Lovett, *op. cit.* (note 60), 100.

69 Loch, *op. cit.* (note 36), 42.

70 ‘Grant of an Increase of Pay’, *op. cit.* (note 48).

71 Loch, *op. cit.* (note 36), 343.

72 Lovett, *op. cit.* (note 60), 100–101.

73 Loch, *op. cit.* (note 36), 341.

74 Arkle, *op. cit.* (note 39), 653; Loch, *op. cit.* (note 36), 341–342.

75 Loch, *op. cit.* (note 36), 341–342.

76
*Ibid.*

77 Arkle, *op. cit.* (note 39), 654.

78 Loch, *op. cit.* (note 36), 41–42.

79
*Ibid.*

80 D.G. Crawford, ‘The Indian Medical Service’, *The Indian Medical Gazette* (May 1907), 192–198: 193.

81 Loch, *op. cit.* (note 36), 344.

82
*Ibid.*, 44.

83
*Ibid.*

84
*Ibid.*, 290.

85
*Ibid.*, 291.

86
*Ibid.*, 292–293.

87
*Ibid.*, 351.

88
*Ibid.*, 328–329.

89
*Ibid.*, 72.

90
*Ibid.*, 71.

91
*Ibid.*, 330.

92
*Ibid.*, 351.

93
*Ibid.*, 350.

94 ‘Adoption of an Uniform Dress for the Members of the Indian Nursing Service’, Government of India (Military Department), Proceedings, January 1900, Nos. 2475-2476. NAI. See Jane Brooks and Anne Marie Rafferty, ‘Dress and Distinction in Nursing, 1860–1939: “A Corporate (as well as Corporeal) Armour of Probity and Purity”’, *Women’s History Review,* 16, 1 (February 2007): 41–57.

95 Loch, *op. cit.* (note 36), 329–330.

96 For an account on similar experience of British nurses in colonial West Africa, see Dea Birkett: ‘The “White Woman’s Burden” in the “White Man’s Grave”: The Introduction of British Nurses in Colonial West Africa’, in Nupur Chaudhuri and Margaret Strobel (eds), *Western Women and Imperialism: Complicity and Resistance* (Bloomington: Indiana University Press, 1992), 177–188.

97
*The India List and the India Office List for 1902* (London: Harrison and Sons, 1902), 209.

98
*Ibid.*, 211.

99 Gorham, *op. cit.* (note 3), 29.

100 Summers, *op. cit.* (note 29), 4.

101 Loch, *op. cit.* (note 36), 98.

102
*Ibid.,* 97–98.

103 Claudia Klaver, ‘Domesticity under Siege: British Women and Imperial Crisis at the Siege of Lucknow, 1857’, *Women’s Writing,* 8, 1 (2001), 21–58: 25; Also see Margaret MacMillan , Women of the Raj: The Mothers, Wives, and Daughters of the British Empire in India (New York: Random House: 2007).

104

Sara Suleri Goodyear , The Rhetoric of English India (Chicago: University of Chicago Press, 1992), 8.

105 See Kumari Jayawardena, *The White Woman’s Other Burden: Western Women and South Asia during British Colonial Rule* (New York: Routledge, 1995), 75; Maneesha Lal, ‘The Politics of Gender and Medicine in Colonial India: The Countess of Dufferin’s Fund, 1885-1888’, *Bulletin of the History of Medicine,* 68, 1 (1994): 29–66; Samiksha Sehrawat, ‘Feminising Empire: The Association of Medical Women in India and the Campaign to Found a Women’s Medical Service’, *Social Scientist,* 41, 5/6 (May 2013): 65–81.

106

Geraldine Forbes , ‘In Search of the ‘Pure Heathen’: Missionary Women in Nineteenth Century India’, Economic and Political Weekly, 21, 17 (1986), 2–8 : 3. Also see Rhonda Anne Semple, *Missionary Women: Gender, Professionalism, and the Victorian Idea of Christian Mission* (New York: Boydell Press, 2003).

107 Loch, *op. cit.* (note 36), 315.

108
*Ibid.,* 329.

109 William St John Brodrick was the British Secretary of State for War. See ‘The Reorganisation of the Army and Indian Nursing Service’, *The Lancet,* October-December (1901), 986; Charlotte Dale argues that the need for nursing expertise during the second Anglo-Boer War (1899-1902), provided the impetus for the formation of the QAIMNS. See Charlotte Dale, ‘Traversing the Veldt with ‘Tommy Atkins’: The Clinical Challenges of Nursing Typhoid Patients During the Second Anglo-Boer War, 1899–1902’, in Jane Brooks and Christine E. Hallett (eds), *One Hundred Years of Wartime Nursing Practices, 1854-1953* (Manchester: Manchester University Press, 2015), 58–78.

110 Please note the difference between QAIMNS and QAMNSI.

111 Lovett, *op. cit.* (note 60), 101.

112 Loch died in 1904. R. A. Betty succeeded Loch as the Chief Lady Superintendent of QAMNSI. See Tooley, *op. cit.* (note 35), 350.

113 Lovett, *op. cit.* (note 60).

114 ‘Grant of an Increase of Pay’, *op. cit.* (note 48).

115 ‘Increase to the Establishment of the Indian Nursing Service’, Government of India (Military Department), Medical Department – Nurses-A, Proceedings, December 1902, Nos. 2033–2036. NAI.

116 Tooley, *op. cit.* (note 35), 350.

117 ‘Grant of an Increase of Pay’, *op. cit.* (note 48).

118 ‘The Reorganisation’, *op. cit.* (note 109), 986.

119

M. R. Truman , Simple Lectures on Nursing for Soldiers in India (Allahabad: The Pioneer Press, 1912).

120
*The India List, op. cit.* (note 97), 211.

121 Truman, *op. cit.* (note 119).

122
*Ibid.*

123 Anne Marie Rafferty argues that education is an important part of professional work and expertise, that plays a pivotal role in shaping an occupational culture and politics. See Anne Marie Rafferty , The Politics of Nursing Knowledge (London: Routledge, 1996).

124 Ashleigh Wadman, ‘Nursing for the British Raj’, *Australian War Memorial* (blog), October 28, 2014, https://www.awm.gov.au/articles/blog/nursing-british-raj. Sisters of the Australian Army Nursing Service (AANS) were sent to India in 1916, to specifically serve in Indian war hospitals that received British and Indian troops wounded at Mesopotamia. The Indian government saved expenditure by employing Australian nurses as they were not granted the allowances availed by the British nurses. For more on AANS sisters, see Samraghni Bonnerjee, ‘“This Country is Rotten”: Australian Nurses in India during the First World War and Their Encounters with Race and Nationhood’, *Australian Journal of Politics & History,* 65, 1 (March 2019): 50–65.

125 Lovett, *op. cit.* (note 60), 101.

126
*Ibid.*, 53.

127 See David Arnold, *Science, Technology, and Medicine in Colonial India*, The New Cambridge History of India, III, 5 (Cambridge: Cambridge University Press, 2000).

128 Lovett, *op. cit.* (note 60), 53.

129
*Ibid.*

130
*Ibid.*, 54.

131
*Ibid.*, 53.

